# Analgesic effects and arthritic changes following intra-articular injection of diclofenac etalhyaluronate in a rat knee osteoarthritis model

**DOI:** 10.1186/s12891-022-05937-y

**Published:** 2022-11-07

**Authors:** Takahito Arai, Miyako Suzuki-Narita, Jun Takeuchi, Ikuko Tajiri, Kazuhide Inage, Yuya Kawarai, Yawara Eguchi, Yasuhiro Shiga, Takashi Hozumi, Geundong Kim, Ryuto Tsuchiya, Takuma Otagiri, Tomohito Mukaihata, Takahisa Hishiya, Noriyasu Toshi, Kohei Okuyama, Soichiro Tokeshi, Takeo Furuya, Satoshi Maki, Yusuke Matsuura, Takane Suzuki, Junichi Nakamura, Shigeo Hagiwara, Seiji Ohtori, Sumihisa Orita

**Affiliations:** 1 Department of Orthopaedic Surgery, Graduate School of Medicine, Chiba University, 1-8-1 Inohana, Chuo-ku, Chiba, 260-8670 Japan; 2 Department of Bioenvironmental Medicine, Graduate School of Medicine, Chiba University, 1-8-1 Inohana, Chuo-ku, Chiba, 260-8670 Japan; 3 Medical Affairs Unit, Seikagaku Corporation, 1-6-1 Marunouchi, Chiyoda-ku, Tokyo, 100-0005 Japan; 4 Department of Orthopaedic Surgery, Kimitsu Chuo Hospital, 1010 Sakurai, Kisarazu-shi, Chiba, 292-8535 Japan; 5 Tertiary Emergency Medical Center (Trauma and Critical Care), Tokyo Metropolitan Bokutoh Hospital, 4-23-15 Kotobashi, Sumida-Ku, Tokyo, 130-8575 Japan; 6 Chiba University Center for Frontier Medical Engineering, 1-33 Yayoi-Cho, CFME Room#B201, Inage-ku, Chiba, 263-8522 Japan

**Keywords:** Diclofenac etalhyaluronate, Rat monoiodoacetate model, Osteoarthritis, Pain

## Abstract

**Background:**

Diclofenac etalhyaluronate (DF-HA) is a recently developed analgesic conjugate of diclofenac and hyaluronic acid that has analgesic and anti-inflammatory effects on acute arthritis. In this study, we investigated its analgesic effect on osteoarthritis, using a rat model of monoiodoacetate (MIA).

**Methods:**

We injected MIA into the right knees of eight 6-weeks-old male Sprague–Dawley rats. Four weeks later, rats were randomly injected with DF-HA or vehicle into the right knee. Seven weeks after the MIA injection, fluorogold (FG) and sterile saline were injected into the right knees of all the rats. We assessed hyperalgesia with weekly von Frey tests for 8 weeks after MIA administration. We took the right knee computed tomography (CT) as radiographical evaluation every 2 weeks. All rats were sacrificed 8 weeks after administration of MIA for histological evaluation of the right knee and immunohistochemical evaluation of the DRG and spinal cord. We also evaluated the number of FG-labeled calcitonin gene-related peptide (CGRP)-immunoreactive(ir) neurons in the dorsal root ganglion (DRG) and ionized calcium-binding adapter molecule 1 (Iba1)-ir microglia in the spinal cord.

**Results:**

Administration of DF-HA significantly improved pain sensitivity and reduced CGRP and Iba1 expression in the DRG and spinal cord, respectively. However, computed tomography and histological evaluation of the right knee showed similar levels of joint deformity, despite DF-HA administration.

**Conclusion:**

DF-HA exerted analgesic effects on osteoarthritic pain, but did not affect joint deformity.

## Background

Osteoarthritis (OA) is a chronic degenerative disease caused by the loss of articular cartilage components in patients worldwide. It is a significant cause of disability-related loss of physical function [1]. Pain is a common symptom of OA, and although its mechanism is unclear, several animal models have been reported, including the anterior cruciate ligament resection [2, 3] and medial meniscectomy [4] models. Monoiodoacetate (MIA) is often used to induce OA because it causes necrosis of chondrocytes when administered in the knee joints of rats, leading to a pathology similar to OA [5, 6].

Previous studies have reported that both inflammatory and neuropathic pain elements and hypersensitivity are present in the animal models of OA [7, 8]. MIA administration which induces OA has been reported to increase the expression of calcitonin gene-related peptide (CGRP) that mediates peripheral nervous system inflammation in the dorsal root ganglia (DRG) [9].

In addition, glial cells, such as microglia in the dorsal horn of the spinal cord, proliferate and change their morphology, and the expression of markers, such as ionized calcium-binding adapter molecule 1 (Iba1) increases that leads to central sensitization [9–11].

Diclofenac etalhyaluronate (DF-HA) is a chemical combination of diclofenac (DF) and hyaluronic acid (HA), a newly developed drug for intra-articular injection in OA that slowly releases DF through hydrolysis in the joint [12]. NSAIDs, such as DF, provide good and rapid analgesia, but are not recommended for frequent or long-term use [13]. HA may improve pain through cartilage protection, anti-inflammation, and intra-articular protection when administered intra-articularly, and may have less immediate, but long-lasting effects [14]. DF-HA may improve joint function and analgesia and decrease systemic side effects because it releases DF slowly by hydrolysis in the joints. Studies suggest that DF-HA promotes the production of high-molecular-weight sodium hyaluronate in human synoviocytes in vitro and may provide potent long-term analgesic effects and improve joint function [15]. Animal studies indicate that DF-HA persists in the joints for approximately 4 weeks after administration and confers analgesic effects on a silver nitrate-induced acute inflammation model [12]. In humans, several clinical trials have reported that joint injections of DF-HA every 4 weeks for up to 52 weeks for osteoarthritis improved pain and did not cause serious adverse events [16–19]. However, in animal studies, no such experimental results have yet been reported for such osteoarthritis models.

An excessive analgesia may promote joint deformity, and studies have reported an association between opioid administration and OA progression [20, 21]. Therefore, this study aimed to investigate the analgesic effect of DF-HA and its effect on joint deformity using an MIA-induced rat knee OA model.

## Methods

### Animals

All animal experimental procedures were reviewed and approved by the Ethics Committee of the Chiba University. All experiments were conducted in accordance with the National Institutes of Health guidelines for the management and use of laboratory animals.

Eight 6-weeks-old male Sprague–Dawley rats (CLEA, Tokyo, Japan), weighing 250–300 g, were used. The rats were kept under an environmentally controlled 12-h light/dark cycle, with a temperature of 21–23℃ and humidity of 45–65%. All rats were provided water and food ad libitum and fed a standard rodent diet (CRF-1; Oriental Yeast Co., Ltd).

### Intra-articular injection of MIA and retrograde neurotracer

Based on previous studies [22], three drugs, 0.15 mg/kg medetomidine (Nippon Zenyaku Kogyo Co., Ltd.), 2.0 mg/kg midazolam (Maruishi Pharmaceutical Co., Ltd.), and 2.5 mg/kg butorphanol (Meiji Seika Pharma. Co., Ltd.) were mixed with 1.45 mL/kg of saline (Otsuka Pharmaceutical Co., Ltd., Tokyo, Japan) and used as anesthesia. The eight rats that had received the anesthetics intraperitoneally were injected with 50 μL of saline and 2 mg of MIA into the right knees using a 27-gauge needle [9, 23].

Four weeks later, the rats were randomly divided into two groups of four rats each: one group was injected with DF-HA (JOYCLU, Seikagaku Corporation) (0.5 mg) (50 μL) into the right knee joint (DF-HA group) and the other group was injected with 50 μL of substrate only (Macrogol 400, an additive adjusted to a pH of 4.8–5.4) without DF-HA (vehicle group).

Seven weeks after MIA administration, the retrograde nerve tracer—1% fluorogold (FG)—and 25 μL of saline were administered to the right knees of all animals.

All rats of both groups were examined for behavioral tests every week and Computed tomography (CT) every 2 weeks. After 8 weeks of administration of MIA injection, namely 4 weeks of DF-HA or vehicle administration, all of them were sacrificed by perfusion fixation under anesthesia. Then, the spinal cord, DRGs and right knee joint of all rats were harvested. The spinal cord and DRGs were used for immunohistochemical staining, and the right knee joints were used for histological evaluation.

### Behavioral test

Using the von Frey assay, mechanical plantar skin sensitivity of all rats in both groups was evaluated every week for 8 weeks after MIA administration. Prior to the test, the condition of each animal was observed, and its weight was measured each time to confirm that there were no health problems. Two groups of four rats each were used. The rats were randomly selected and acclimatized to the test chamber for 1 h before testing (blinded to the experimenter). Baseline thresholds were tested before MIA administration. The von Frey filament (monofilament kit; Smith & Nephew) was applied for 4 s or until the lower limb was withdrawn (whichever occurred first), and the 50% paw withdrawal threshold (PWT g) was calculated [24]. Stimulus intensity ranged from 1 to 60 g, corresponding to 4.08, 4.17, 4.31, 4.56, 4.74, 4.93, 5.07, 5.18, 5.46, and 5.88. Five consecutive stimulations were performed using the up-down method [25], starting from the lowest filament that elicited a positive response in each animal. Threshold values were calculated based on the filament thickness, average spacing, and pattern of responses. Hind limb plantar mechanical hypersensitivity was assessed using a wire mesh observation cage. Data are presented as 50% PWT ± standard mean error for each group.

### Immunohistochemical analyses of CGRP and Iba1

As mentioned above, according to a previous study [26], the L3, L4, and L5 level right DRG and lumbar spinal cord of all the rats in both groups were harvested 4 weeks after the administration of DF-HA or vehicle, namely 8 weeks after administration of MIA. The DRG and spinal cord specimens were immersed overnight in phosphate-buffered paraformaldehyde.

Specimens were then stored in 0.01 M phosphate-buffered saline (PBS) containing 20% sucrose for 20 h at 4 ℃ and frozen in liquid nitrogen. The DRG and spinal cord were sliced into 10-μm-thick slices using a cryostat (CM3050S, Leica Microsystems). The sections were mounted on slides coated with poly-L-lysine. The DRG sections were treated with a nonspecific blocking solution of 0.3% Triton X-100 mixed with 3% skim milk, bovine serum albumin, and PBS for 90 min at 20℃. The sections were then incubated with anti-CGRP rabbit antibody (1:1,000 dilution; chemicon Temecula) for 20 h at 4℃, and then incubated with Alexa Fluor 488-conjugated goat anti-rabbit IgG (1:1,000 for CGRP immunization Molecular Probes).

The spinal cord sections were incubated with anti-Iba1 rabbit antibody (1:1,000 dilution; Chemicon Temecula) for 20 h at 4℃, and then incubated with Alexa Fluor 488-conjugated goat anti-rabbit IgG (1:1,000 for Iba1 immunization, Cat# 019–19,741; Wako, Osaka, Japan).

After each step, the sections were washed three times with PBS. Immunostained sections were observed under a microscope BZ-X810 (KEYENCE, Japan).

The number of neurons labeled with FG-alone and those marked with FG and CGRP were randomly counted at 10 locations every 0.45 mm^2^ in each DRG at 400 × magnification. The percentage of CGRP-immunoreactive (ir) neurons in the FG-labeled neurons was then determined.

Counting grids were used to measure Iba1-ir neurons every 0.1 mm^2^ of the dorsal horn of the spinal cord.

Both CGRP-ir DRG neurons and Iba-positive cells in spinal cord were completely blinded to count by two orthopedic surgeons with experience in basic experiments.

### Histopathological findings

Samples were collected from all groups for histological evaluation. The rats were anesthetized intraperitoneally with 0.15 mg/kg medetomidine, 2.0 mg/kg midazolam, and 2.5 mg/kg butorphanol. Further, 0.9% saline was perfused percutaneously, followed by 500 mL of 4% paraformaldehyde mixed with phosphate buffer (0.1 M, pH 7.4). The soft tissues around the right knee joint were excised. Each specimen was dehydrated twelve hours in U.I. demineralization solution A (Yuaikasei CO., LTD, Amagasaki, Hyogo, Japan), neutralized with 5% sodium sulfate, and then cut. The specimens were dehydrated and paraffin-embedded using Tissue-Tek V. I. P. 6 AI (Sakura Finetek Japan Co., LTD, Tokyo, Japan). From each prepared block, 10-μm serial sections were made at the center and intercondylar area of the knee joint using a sliding microtome LS113 (YAMATO-KOHKI Industrial Co., LTD, Asaka, Saitama, Japan). Twelve sections from each group were stained with hematoxylin–eosin and safranin O (three sections per animal).

OA was evaluated using the histopathology scoring system of the Osteoarthritis Research Society International (OARSI) [27]. The OARSI score is calculated by multiplying the Stage and Grade scores. Grade 0 is normal, grade 1 preserves the articular surface, but cartilage tissue changes such as chondrocyte proliferation occur, grade 2 shows localized breakdown of cartilage surface continuity, grade 3 shows progressive vertical fissuring, grade 4 shows delamination of the cartilage surface, grade 5 shows degeneration extending to the subchondral bone, and grade 6 shows deformity of the joint as a result of microfracture repair. Stage 0 has no OA findings, stage 1 has less than 10% of the joint surface, stage 2 has 10–25%, stage 3 has 25–50%, and stage 4 has more than 50%. Two orthopedic surgeons with experience in basic experiments blindly assessed and scored the samples.

Each sample was evaluated for OA changes in depth (grading) and width (staging), and the scores for depth and width were multiplied. The mean scores for each group were compared between groups.

### Radiographical findings

CT of the right knee of rats in both the groups was performed every 2 weeks after MIA administration. The rats were anesthetized by inhalation of sevoflurane, and imaging was performed using a CT Lab GX (Rigaku, Tokyo, Japan) with a FOV of 45 mm and an imaging time of 18 s.

Coronal cross-sectional images parallel to the bony axis of the tibia and through the center of the articular surface were produced on CT and evaluated using the Larsen classification [28], commonly applied in rheumatoid arthritis. Two blinded orthopedic surgeons evaluated and scored the sections according to the degree of joint deformity. The mean scores of each group were compared between the groups.

### Statistical analysis

Statistical analyses were performed using GraphPad Prism version 9 (GraphPad Software). PWT, OARSI score, percentage of CGRP-positive FG-labeled neurons in the DRGs, and the number of microglia in the right dorsal horn of the spinal cord were compared between the two groups using Mann–Whitney U test. Statistical significance was set at *P* < 0.05.

## Results

### Behavioral test

**Fig. 1:**
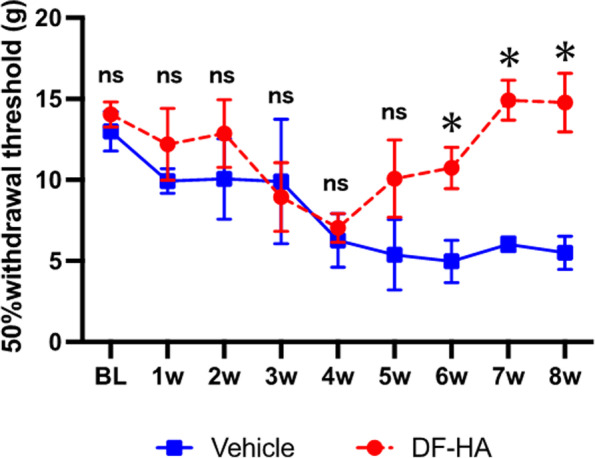
Effect of DF-HA on the pressure withdrawal threshold applied to one posterior ipsilateral limb Changes in hyperalgesia are measured using a von Frey filament and expressed in grams (g) as a 50% paw withdrawal threshold. Behavioral tests are performed before MIA administration (BL). Further, 2 mg of MIA is administered, and the von Frey test is performed weekly for 8 weeks. DF-HA (DF-HA group) or vehicle (vehicle group) is administered at week 4 and FG is administered at week 7 after the test. Lower threshold values indicate enhanced hyperalgesia. Data are expressed as the mean ± standard error of four animals per group

In the von Frey test, both the vehicle and DF-HA groups showed a decreased pain threshold after MIA administration, with no significant difference between the two groups. After drug administration, the DF-HA group showed a significant improvement in irritability from the second to the fourth week after treatment than vehicle group (*P* < 0.05; Fig. 1).

### Immunohistochemical expression of CGRP and Iba1

**Fig. 2:**
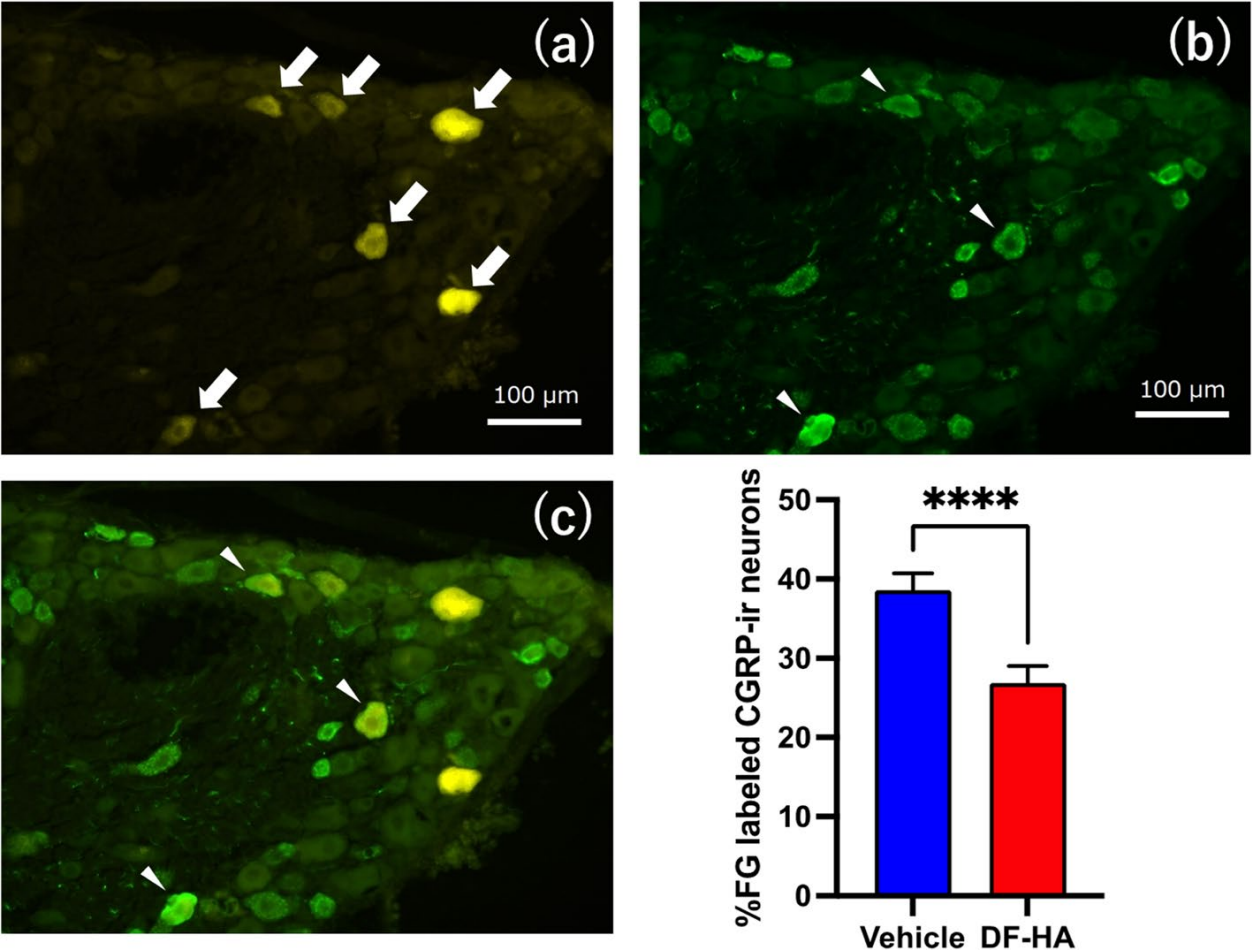
Fluorescence photomicrographs of the DRG after administration of DF-HA or vehicle for 4 weeks **a** FG-labeled DRG neurons, **b** CGRP-immunoreactive (ir) DRG neurons, and **c** overlaid picture **a** on **b**. All photomicrographs are from the same section. White arrows in the photomicrograph of **a** indicate FG-labeled DRG neurons, and white arrowheads in the photomicrograph of **b** and **c** indicate FG-labeled CGRP-ir DRG neurons. The proportions of FG-labeled CGRP-ir neurons in the DF-HA groups are significantly higher than those in vehicle group (*n* = 4 rats per group)

**Fig. 3:**
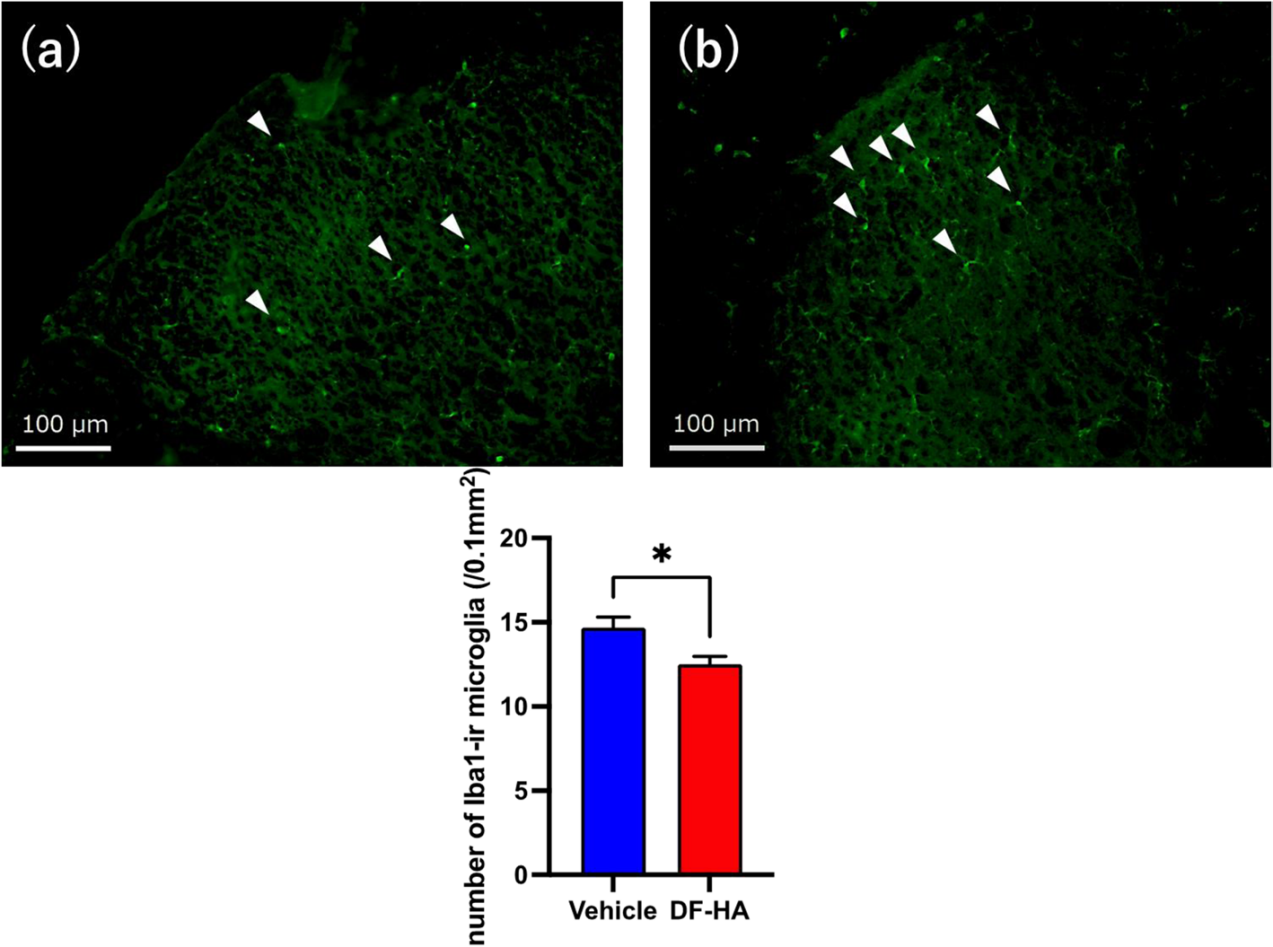
Fluorescence photomicrographs of the spinal cord after administration of DF-HA or vehicle for 4 weeks **a** and **b** Representative fluorescent photomicrographs of the right dorsal horn of the spinal cord. Scale bars, 100 μm. White arrowheads indicate Iba1-immunoreactive (ir) microglia in **a** DF-HA and **b** vehicle groups. The number of Iba1-ir microglia is significantly higher in the vehicle group than in DF-HA group

The DF-HA group had a significantly lower percentage of FG-labeled CGRP-ir cells in the DRGsL3, L4 and L5 than those in vehicle group (*P* < 0.01; Fig. 2). The number of Iba1-ir cells in the right dorsal horn of the spinal cord was significantly lower in the DF-HA group than in vehicle group (*P* < 0.05; Fig. 3).

### Histopathological findings

**Fig. 4:**
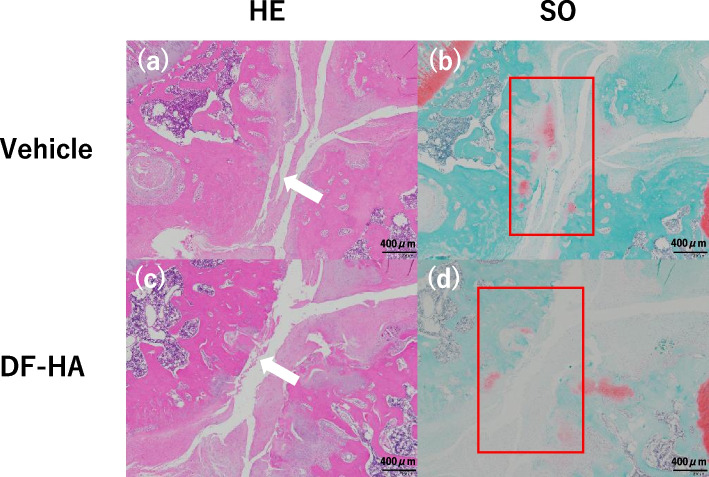
Histopathological images of the rat’s knee in the vehicle and DF-HA groups **a** and **b** Vehicle group, and **c** and **d** DF-HA group. Scale bars, 400 μm. **a** and **c** Hematoxylin–eosin staining. **b** and **d** Safranin O (SO) staining. **a** and **c** White arrows indicate significant cartilage loss in both groups. **b** and **d** Little cartilages are stained red with SO. **b** and **d** Red-stained areas are irregularly fragmented and distributed deep in the remaining cartilage layer (sections surrounded by a red frame)

**Fig. 5:**
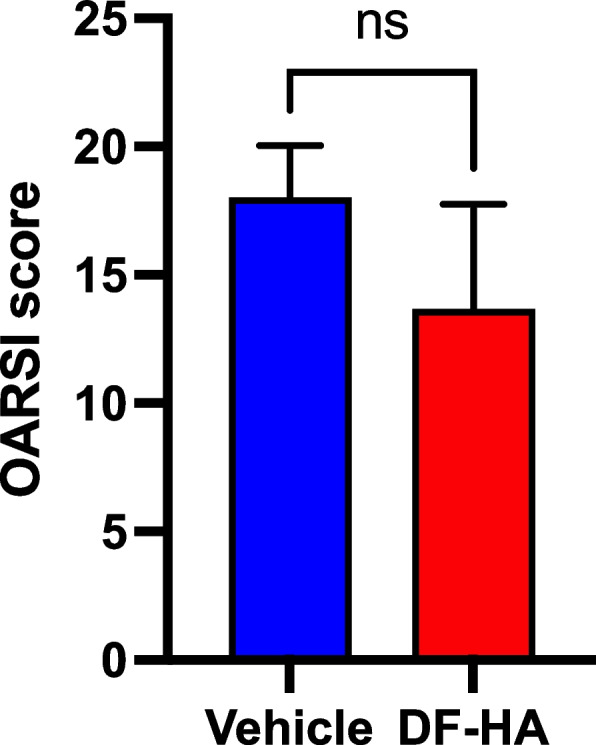
Graphs of OARSI score in each group after 4 weeks of DF-HA or vehicle administration The mean score of the vehicle group is slightly higher than that of DF-HA group (*P* > 0.05)

Both groups had progressive OA changes (Fig. 4). The OARSI score was slightly lower in the DF-HA group than in vehicle group, but there was no significant difference between the two groups (Fig. 5).

### Radiographical findings

**Fig. 6:**
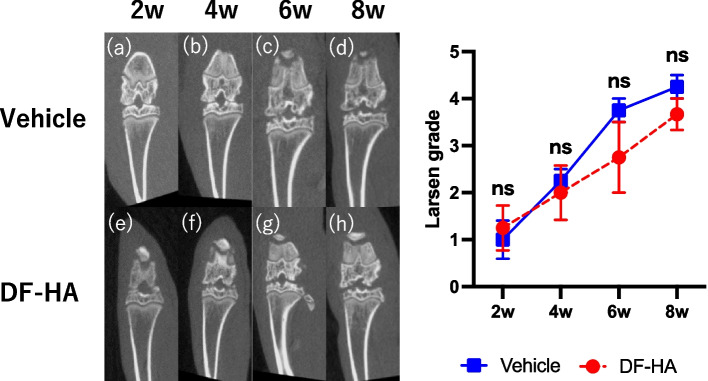
CT images of the right knee after intra-articular injection of MIA every 2 weeks **a–d** Vehicle and **e–h** DF-HA groups. The graph shows the transition of the mean Larsen grade of each group. Joint degeneration tends to progress with time (*P* > 0.05)

Progression of the joint deformity was observed in both the groups; however, there was no significant difference at any time point (Fig. 6).

## Discussion

This is the first study to demonstrate the effect of DF-HA treatment on the behavioral induction of osteoporotic pain, sensory neurons, and OA in a rat model of MIA-induced knee OA. In the present study, 2.0 mg of MIA administered to the knee joint of rats led to significant MIA-induced cutaneous hind paw hypersensitivity to mechanical stimuli and OA changes. In addition, intra-articular injection of DF-HA caused significant improvement in cutaneous hind paw hypersensitivity to mechanical stimuli and attenuated CGRP-ir DRG neurons and Iba-1 positive cells. However, there was no significant difference in the osteoarthritic changes between the DF-HA and vehicle groups.

In the 2.0 mg MIA rat knee OA model used in this study, significant MIA-induced cutaneous hind paw hypersensitivity to mechanical stimuli, increased CGRP-ir DRG neurons and MIA-induced OA changes have been previously reported [9], and the model is considered a valid model for knee OA.

Since this is the first study in which DF-HA was administered to a rat MIA model, it was necessary to use a dose that would definitely induce joint deformity. In addition, since it was required to compare not only arthropathic changes but also pain behavior evaluation, we decided to use a dose of 2.0 mg MIA. This dose has been reported in previous animal studies using rats to reliably induce arthropathic changes and show significant results in pain behavior. We also considered MIA 2.0 mg, which causes severe joint degeneration, to be appropriate because, in Japan, in clinical practice, intra-articular injection of HA and DF-HA is often used for patients with moderate to end-stage OA as well as for those with mild OA.

However, DF-HA may exert better analgesic and joint-protective effects in patients with mild OA. Yoh et al. [29] reported that 0.25 mg or 0.5 mg MIA intra-articular injection to hip in rats induced mild OA this year, moreover, Kanno et al. [26] also reported arthropathic changes and pain behavior assessment in a rat model of hip osteoarthritis at 0.5 mg MIA. We believe it is necessary to compare the effects of DF-HA with those of low-dose MIA administration models such as 0.25 mg and 0.5 mg in the future.

In a previous study, radiolabeling showed that DF-HA remained in the joints for up to 4 weeks post-administration and released DF continuously [30]. In addition, a study [12] in which DF-HA was administered after inducing arthritis with silver nitrate in the knees of rabbits suggested that the DF concentration in the synovium remained > 10 ng/g and significantly reduced swelling from the day after its administration until 4 weeks later. In the present study, MIA-induced cutaneous hind paw hypersensitivity to mechanical stress was significantly improved in the DF-HA group than that in vehicle group from 2 to 4 weeks after administration. These results suggest that intra-articular injection of DF-HA improved pain and was sustained for up to 4 weeks after administration.

In this study, considering the survival period of FG, intra-articular injection of FG was performed at week 7 of MIA administration, but the possibility of influence on subsequent pain behavior cannot be ruled out. In the future, it will be necessary to devise the timing of FG administration, such as administering MIA and FG at the same time and conducting further long-term follow-ups.

A previous study reported that intra-articular administration of MIA enhances cyclooxygenase2 (COX2) and interleukin1β (IL-1β) expression in chondrocytes [31]. DF-HA continuously released DF that suppressed the production of prostaglandin E2 [12, 32], a typical inflammatory cytokine, by inhibiting COX, thereby reducing pain. These mechanisms suggest that DF improves pain in the MIA-induced rat knee OA model.

Immunohistochemical staining showed that the DF-HA group had a significantly decreased percentage of FG-positive and CGRP-positive DRG cells innervating the knee joint than those in vehicle group. The secretion of local pro-inflammatory cytokines contributes to increased CGRP expression, leading to neurogenic inflammation and hypersensitivity.

The afferent nerve fibers in the rat knee joint have been reported to be localized to the L3, L4, and L5 [33, 34]. Increased levels of CGRP in the DRG of L3, L4, and L5 indicated acute inflammatory pain in the knee of an MIA-induced OA rat model [35–37]. In addition, the DRG neurons are considered responsible for acute inflammatory pain [38].

Thus, pain-related characteristics of an MIA-induced rat OA model may originate from an inflammatory pain state induced by local inflammation initiated by inflammatory cytokines. Therefore, the present study indicated that local inflammation occurred in the knee of an MIA-induced OA rat model, and that CGRP expression in the DRG was suppressed by DF-HA injection that suggested that DF-HA administration reduced acute inflammatory pain. However, as there was no control group in this study, it is unclear how many CGRP-ir DRG neurons were elevated after MIA injection in the vehicle group than in normal group. Future studies should include a control group.

In this study, the number of Iba1-positive cells, a marker of microglia in the dorsal horn of the spinal cord, was also significantly reduced in the DF-HA group than that in vehicle control. Pain in OA is a complex mechanism; however, it may primarily involve inflammatory pain and, when prolonged, a neuropathic pain component. Moreover, in the MIA-induced model, pain may become chronic due to central sensitization [38, 39].

It has been shown that 2.0 mg of MIA administered intra-articulately causes significant axonal damage to DRG cells innervating the skin and other parts of the hind leg, in addition to joint deformities [33]. Namely, neuropathic pain component may have been involved in the present results with MIA 2.0 mg.

In this study, we didn't evaluate the nature of the pain, and immunostaining of DRGs only evaluated inflammatory pain using CGRP. Therefore, neuropathic pain was not discussed. Therefore, future studies should include evaluation of ATF-3, a neuropathic pain marker in DRGs, and microglial activity using OX42 in the dorsal horn of the spinal cord to focus on the neuropathic pain component when we use 2.0 mg MIA injection.

Level of COX is upregulated in macrophages and Schwann cells around nerve injury [40], and COX inhibitors can interfere with myelin debris signals that negatively affect regeneration and may assist in nerve regeneration in rats [41, 42]. DF-HA may also decrease microglial expression by regenerating peripheral nerves and inhibiting sensitization of the central nervous system. Therefore, DF-HA may be effective against acute inflammatory and chronic pain; however, further validation is needed.

Pathologically and radiographically, DF-HA was ineffective in improving or inhibiting the progression of joint deformity; in contrast, there was no progression of deformity due to excessive analgesia. As Kanno et al. [26] reported, drugs with strong analgesic effects such as tramadol risk worsening arthropathy, but the results of this study showed that DF-HA reduced pain but did not worsen arthropathy. This may be because HA has a protective effect on joints and promotes the synthesis of proteoglycans and glycosaminoglycans [43].

In this study, we used the Larsen classification for CT image evaluation because CT is more convenient than X-ray at our facility and that it is more accurate to evaluate the articular surface of rats than X-ray. The Kellgren–Lawrence classification [44] (K–L classification) on X-rays is generally used for evaluating human patients with OA. However, in CT [45], particularly in rats, MIA injection causes bone loss, and increases trabecular [46] and tibial subchondral plate thickness, but this is not a well-established evaluation. As the MIA-induced rat model simulates OA by inducing necrosis of chondrocytes, we considered the Larsen classification to be more suitable for the evaluation than the K–L classification. Considering the characteristics of CT, it is possible to provide detailed data on the evaluation of the bone itself, such as bone volume/tissue volume (BV/TV), trabecular number (Tb.N), trabecular thickness (Tb.Th), trabecular separation (Tb. Sp), we would like to evaluate the bone using each parameter with CT.

This study has some limitations. First, we failed to include a control group. In previous studies, it has been reported that 2.0 mg MIA induces obvious arthropathic changes, and in this study, CT showed obvious arthropathic changes, so we judged that it is appropriate as a rat knee arthropathy model induced by MIA. However, the lack of a control group is a major limitation of this study, and it will be necessary to provide one in future studies. Second, as the synovial membrane and cartilage in the knee joint were not investigated, we could not confirm whether the reduction in inflammatory cytokines decreased the pain. Third, the von Frey test was the only behavioral test used to evaluate pain. Therefore, it is necessary to perform behavioral assessments for musculoskeletal pain, such as measuring hind limb weight bearing and the catwalk test in the future. Finally, the follow-up period was short, and DF-HA was administered only once. Thus, a long-term study with adequate administration of DF-HA is required in the future.

## Conclusion

Intra-articular injection of DF-HA provided an analgesic effect without worsening joint deformity in the rat knee MIA model.

## Data Availability

The dataset supporting the conclusions of this study are available from the corresponding author on reasonable request.

## Abbreviations

DF-HA: Diclofenac etalhyaluronate
MIA: Monoiodoacetate
FG: Fluorogold
CGRP: Calcitonin gene-related peptide
DRG: Dorsal root ganglion
Iba1: Ionized calcium-binding adapter molecule 1
CT: Computed tomography
OA: Osteoarthritis
DF: Diclofenac
HA: Hyaluronic acid
Ir: Immunoreactive
PWT: Paw withdrawal threshold
PBS: Phosphate-buffered saline
OARSI: Osteoarthritis Research Society International
COX2: Cyclooxygenase2
IL-1β: Interleukin1β
K-L: Kellgren–Lawrence
